# Epistemic justice in educational assessment: decolonizing measurement through indigenous knowledge systems and Afrocentric paradigms

**DOI:** 10.3389/frma.2026.1903343

**Published:** 2026-09-09

**Authors:** Moses Mhide Kpum, Velisiwe Gasa

**Affiliations:** Department of Educational Foundations, College of Education, University of South Africa (UNISA), Pretoria, South Africa

**Keywords:** Afrocentric pedagogy, culturally responsive assessment, decolonization of assessment, educational assessment, epistemic justice, inclusive evaluation practices, indigenous knowledge systems

## Abstract

Educational assessment in many African contexts continues to be shaped largely by Eurocentric traditions of measurement that emphasize standardization, objectivity, and uniformity. While these approaches have contributed to the development of formal education systems, they have also tended to overlook or marginalize Indigenous Knowledge Systems (IKS), Afrocentric perspectives, and community-based ways of knowing. This raises important questions about whose knowledge is recognized and valued within existing assessment practices. This paper examines the issue of epistemic justice in educational assessment, with particular attention to how dominant assessment models may exclude culturally grounded forms of knowledge. Drawing on decolonial perspectives and Afrocentric scholarship, the study adopts a qualitative, conceptual approach to critically reflect on the assumptions underpinning current assessment frameworks in Anglophone, and predominantly Southern African, higher education systems. It argues that assessment is not merely a technical process but is shaped by broader historical, cultural, and political influences. The paper explores how indigenous ways of knowing, oral traditions, experiential learning, and communal validation are often insufficiently captured within conventional assessment formats. In response, it proposes more inclusive approaches that recognize multiple forms of knowledge and ways of demonstrating competence, including culturally responsive assessment practices and participatory, community-informed methods of evaluation, illustrated with documented institutional cases, and considers the implications of emerging digital and AI-based assessment tools, noting the risk that existing biases may be reproduced and amplified if such technologies are not carefully adapted to local contexts. The study contributes to ongoing discussions on decolonization and knowledge justice by highlighting the need to rethink assessment in ways that are more responsive to African realities and epistemologies, while remaining explicit about the geographic and linguistic boundaries of its own evidence base.

## Introduction

The question of what counts as knowledge, and who has the authority to produce and validate it, lies at the heart of educational assessment. In African higher education institutions, this question carries particular historical weight. The assessment frameworks that govern how student learning is evaluated were, in their foundational architecture, designed within and for Eurocentric educational systems. They carry within them assumptions about the nature of knowledge, its separability from context, its expression through standardized written forms, its validation through individual rather than communal demonstration, that are not universally shared and that do not map cleanly onto the rich epistemic traditions of African peoples (Ndofirepi and Gwaravanda, 2019; Santos, 2018).

The consequences of this mismatch are not merely academic. When assessment systems fail to recognize the knowledge and capabilities that learners bring from their cultural and community contexts, they do not simply leave certain competencies unmeasured; they actively communicate that those competencies do not count. This is, in Miranda Fricker's (2007) terms, an epistemic injustice—a wrong done to someone in their capacity as a knower. Fricker identified two primary forms of such injustice: testimonial injustice, in which a speaker's credibility is unfairly diminished due to prejudicial identity assumptions, and hermeneutical injustice, in which a person's experience or knowledge cannot be adequately understood or articulated because the dominant interpretive frameworks do not accommodate it. Both forms are clearly operative in assessment contexts that privilege written, standardized, English-medium demonstration of knowledge while marginalizing oral, communal, experiential, and vernacular forms of knowing.

The urgency of addressing these questions has grown substantially in recent years. The movement to decolonise higher education, energized in the South African context by the #RhodesMustFall and #FeesMustFall campaigns of 2015–2016 and sustained across the continent by a generation of scholars committed to epistemic freedom, has placed curriculum and assessment transformation at the center of institutional debates (Ndlovu-Gatsheni, 2021). Yet it is notable that, while considerable attention has been paid to the decolonization of curricula and pedagogical approaches, the specific domain of educational assessment has received comparatively less systematic treatment. Assessment reform tends to lag behind curriculum reform, in part because of the entrenched institutional and accreditation frameworks that govern how student performance is evaluated and credentialled. A recent systematic review of empirical scholarship on decolonial assessment in higher education confirms this lag: of the studies that meet basic empirical criteria, the great majority originate from South Africa and the United States, with almost no representation from the rest of the African continent (Madonda and Jita, 2026). This paper addresses that specific gap—theoretical engagement with the epistemics of assessment injustice, read primarily through Southern African and Anglophone African scholarship.

A note on the scope of this argument is warranted at the outset, in response to a legitimate concern about generalization. This paper speaks of “African higher education” and “African students” for economy of expression, but its evidentiary and scholarly base is concentrated in Anglophone, and predominantly Southern African, sub-Saharan contexts. The continent's linguistic, colonial, and institutional diversity is considerable: Francophone West African evaluation systems, for instance, have been shown to negotiate stakeholder inclusion and epistemic authority in ways that only partially overlap with Anglophone Southern African patterns (Dossou Kpanou et al., 2025), and Lusophone and North African higher education systems carry distinct colonial histories, language policies, and assessment traditions that this paper does not directly examine. Where the argument makes claims that plausibly hold across these varied contexts, it does so cautiously and flags this as a claim requiring further comparative testing rather than an established finding; where it draws on evidence specific to Southern Africa, it says so explicitly. Readers working in Francophone, Lusophone, or North African higher education are invited to treat the paper's claims as a starting hypothesis for their own contexts rather than an automatic transfer.

The paper proceeds through several interrelated stages. It begins by locating the problem theoretically within the broader frameworks of epistemic injustice, decolonial thought, and Afrocentric scholarship. It then describes the methodology that underpins the conceptual analysis. It maps the specific mechanisms through which conventional assessment practices in African higher education reproduce epistemic injustice. It explores what indigenous and Afrocentric epistemologies offer as alternatives, and how those alternatives might be translated into assessment practice. It addresses the particular challenge posed by the growing deployment of digital and AI-based assessment tools. And it draws these threads together into a set of principles for epistemically just assessment, including a discussion of how such principles might be reconciled with the practical demands of international accreditation and graduate mobility. Throughout, the paper insists that this is not merely a technical project of assessment reform but a political and ethical one, one that requires institutions to reckon honestly with the colonial inheritance that continues to shape how knowledge is evaluated in African universities. To ground the argument in more than theory, the discussion draws at several points on documented institutional cases, among them the redesign of history assessment at a South African university (Godsell, 2021; Godsell et al., 2024), group portfolio assessment across South African universities (Fouche et al., 2021), the integration of Indigenous Knowledge Systems into teacher education curricula in South Africa (Makokotlela and Gumbo, 2025), and the structural embedding of Indigenous Knowledge Systems at the African Rural University in Uganda (Maali, 2025). These cases are illustrative rather than representative, and are discussed as such.

## Theoretical framework: epistemic justice, decoloniality, and Afrocentricity

### Fricker's epistemic injustice and its educational dimensions

Fricker's (2007) framework of epistemic injustice has become an important conceptual resource for scholars examining the politics of knowledge in educational settings. Her identification of testimonial injustice—the systematic deflation of a speaker's credibility due to prejudicial assumptions about their identity—applies directly to educational assessment contexts in which students from African, rural, or working-class backgrounds find their knowledge claims, modes of argument, and ways of knowing treated as epistemically inferior by assessors whose evaluative frameworks are calibrated against Eurocentric norms. The knowledge that a student possesses may be entirely genuine and academically relevant, yet if it is expressed in forms that fall outside the expected conventions, through oral elaboration, through reference to community knowledge, through vernacular conceptual frameworks, it is liable to be evaluated as insufficient or misaligned with the assessment criteria.

Hermeneutical injustice, Fricker's (2007) second category, is in many ways more structurally significant for assessment reform. It occurs when there is a gap in the collective interpretive resources available to a community of evaluators, such that forms of knowledge or experience that are legitimate within one epistemic tradition simply cannot be rendered visible through the evaluative instruments of another. In African higher education, this gap is systematic and historically produced. Curricula and assessment frameworks that were designed to reproduce Eurocentric knowledge do not merely disadvantage students who know things differently; they render entire traditions of knowing hermeneutically invisible, that is, incapable of being registered within the evaluation framework regardless of their genuine epistemic merit (Ndofirepi and Gwaravanda, 2019). Dotson's (2012) concept of contributory injustice extends Fricker's framework to include cases in which certain groups are actively prevented from contributing to the expansion of shared interpretive resources—a condition that is particularly pertinent to universities where the authority to define assessment standards remains concentrated among scholars trained in Western academic traditions.

### Decolonial epistemology: Santos, Mignolo, and the ecology of knowledges

Santos (2018) argues that the global expansion of Western modernity produced not only political and economic domination but epistemicide—the systematic destruction of the epistemic traditions of colonized peoples through the imposition of European knowledge as the universal standard of rationality and truth. Within this framework, the modern university is not a neutral space of knowledge transmission but an institution that has historically participated in the perpetuation of epistemic hierarchies, treating indigenous and non-Western knowledge traditions as superseded, primitive, or merely supplementary to the main body of legitimate scholarship.

Santos's (2018) concept of the “ecology of knowledges” offers a counter-proposal: rather than accepting a single epistemological framework as authoritative, an ecology of knowledges recognizes that different knowledge traditions have different but equally legitimate contributions to make to understanding the world. Applied to assessment, this concept implies that evaluation frameworks must be capable of recognizing and crediting forms of knowledge that are legitimate within multiple epistemological traditions—not only those that conform to the conventions of Western academic writing. Walter Mignolo's (2012) concept of “border thinking” complements this by pointing to the creative epistemological work that occurs at the intersection of multiple knowledge traditions, work that is rendered invisible by assessment systems that recognize only the products of engagement with canonical academic literature.

Ndlovu-Gatsheni's (2021) concept of the “cognitive empire” provides an African-centered articulation of the same epistemological problem. He argues that the global structures of knowledge production continue to be organized around the epistemic authority of the Global North, such that African scholars and institutions are positioned as consumers rather than producers of authoritative knowledge. The African university, in this analysis, remains in many respects a site of epistemic subjugation—a place where knowledge hierarchies inherited from colonialism are reproduced through curricula, assessment, and accreditation systems that continue to privilege Northern epistemologies. Assessment reform is not peripheral to but constitutive of the project of epistemic liberation that decolonial scholarship demands.

### Afrocentricity as an epistemological resource for assessment reform

Afrocentric scholarship, developed most influentially by Asante (1987) and Asante (2003), provides a further conceptual resource for rethinking assessment in African contexts. Afrocentricity insists that African people should be positioned as subjects rather than objects of knowledge, as agents who produce, validate, and transmit knowledge from the vantage point of their own historical, cultural, and philosophical traditions rather than as recipients of knowledge produced elsewhere and evaluated against external standards. Asante's concept of “centring” of grounding analysis in African experience and ontology offers a paradigmatic alternative to the Eurocentric assumption that universal knowledge is produced at a single epistemological center and disseminated outward. This paper treats Afrocentricity, Ubuntu philosophy, and Indigenous Knowledge Systems as identifying dominant tendencies within diverse and internally contested African intellectual traditions, not as claims that these traditions are internally homogeneous or that all African communities share identical epistemological commitments; the same caution applies to the discussion of oral tradition and communal validation later in the paper.

For assessment, the Afrocentric paradigm implies a fundamental reorientation of the questions that evaluation asks. Rather than asking whether African students can demonstrate competence in terms defined by European intellectual traditions, it asks how African students can be supported in demonstrating the full range of their capabilities, including capabilities rooted in indigenous knowledge, communal wisdom, oral tradition, and the lived experience of African communities, within frameworks that recognize and credit these capabilities as academically legitimate. This requires not only the diversification of assessment formats but a reconceptualization of what assessment is for: not the ranking of students against a fixed norm derived from dominant cultural practices, but the recognition and development of the multiple forms of intelligence and knowledge that students bring with them to higher education.

## Methodology

### Conceptual research design

This paper adopts a qualitative, theoretically informed conceptual research design anchored in critical theoretical analysis. Conceptual research involves the systematic examination, clarification, and development of theoretical constructs and their interrelationships, with the aim of generating insights that advance scholarly understanding and inform practice (Jabareen, 2009). It is a well-established methodology in educational research, particularly in contexts where the primary aim is to identify the assumptions embedded in existing frameworks and to propose alternative conceptual orientations. This study employs conceptual analysis not to resolve an empirical question about what is happening in particular classrooms or institutions but to interrogate the theoretical and philosophical foundations of assessment practice and to articulate a more epistemically just alternative. Consistent with reviewer guidance, we characterize the paper explicitly as a theoretically informed argument paper that follows the procedural discipline of conceptual analysis (Jabareen, 2009) rather than the reporting standards of a formal systematic review; the illustrative institutional cases discussed below (Section 4 and 5) are used to ground and test the argument, not to establish generalizable empirical findings.

The choice of conceptual methodology is appropriate to the nature of the research problem. The epistemic injustice that this paper examines is, at its core, a problem of inadequate theoretical frameworks: frameworks that have naturalized Eurocentric assumptions about knowledge to such a degree that they have become invisible as assumptions rather than appearing as what they are—historically produced, culturally situated, and politically consequential choices about whose knowledge counts. Addressing this problem requires precisely the kind of theoretical work that conceptual research makes possible: the articulation of alternative frameworks, the examination of their implications, and the identification of the institutional and pedagogical changes they would require. As Chilisa (2020) emphasizes, indigenous and decolonial research paradigms legitimately work through conceptual and theoretical modes that are attuned to the knowledge traditions and epistemological frameworks of the communities whose education is at stake.

### Sources and analytical approach

The conceptual analysis draws on a purposively selected corpus of scholarly literature spanning educational assessment theory, decolonial and postcolonial epistemology, Afrocentric scholarship, indigenous knowledge systems, and the philosophy of education. Literature was identified through systematic searches of the Scopus, Web of Science, and Google Scholar databases conducted between February 2026 and June 2026, with a final citation check completed in July 2026, using search terms including “epistemic justice,” “decolonization and assessment,” “Indigenous Knowledge Systems and education,” “Afrocentric pedagogy,” “culturally responsive assessment,” “African higher education and knowledge,” and “AI assessment and Global South.” Priority was given to peer-reviewed publications from 2016 to 2026, with classical theoretical texts, particularly Fricker (2007), Asante (1987), and Santos (2018), included where their foundational contributions are indispensable to the argument.

Sources were included where they (a) were published in a peer-reviewed journal, edited volume, or reputable institutional/policy report; (b) were available in English or in a translation the authors could verify against the original; (c) engaged substantively with epistemic justice, decolonization, Indigenous Knowledge Systems, Afrocentric or Ubuntu philosophy, or assessment/evaluation practice in African or comparable Global South contexts; and (d) fell within the 2016–2026 window, with the exception of foundational theoretical texts cited above. Sources were excluded where they addressed decolonization or assessment only in passing, where they could not be verified against a traceable publication record, or where they concerned school-level (rather than higher education) assessment, unless directly relevant to a specific illustrative point. Gray literature and institutional reports (e.g., African Union and UNESCO policy documents) were included only where no peer-reviewed equivalent was available and the document was an official publication of the issuing body.

Sources were selected on the basis of their theoretical significance, their contribution to debates on decolonization and epistemic justice in African higher education specifically, and their representation of scholarly perspectives from African contexts alongside more internationally circulated scholarship. The analysis proceeded through a process of thematic mapping, following the reflexive thematic analysis procedure described by Braun and Clarke (2022): close reading and open coding of the corpus, iterative clustering of codes into candidate themes, and discussion between the author and co-author to review, refine, and name the themes against the paper's guiding questions: identifying the key mechanisms through which conventional assessment practices reproduce epistemic injustice, clarifying what Afrocentric and indigenous epistemic frameworks offer as alternatives, and synthesizing these insights into a principled account of epistemically just assessment. The paper's conclusions are offered as theoretical propositions that require empirical testing and contextual adaptation across the diverse institutional settings of African higher education.

### Positionality

The authors write as researchers and academics based at a South African institution with sustained scholarly engagement in questions of educational equity, assessment theory, and African education systems. This positionality is both an asset and a source of limitation: it affords a grounded understanding of the institutional realities and scholarly debates that characterize higher education in the South African context, while inevitably reflecting a particular vantage point within the broader diversity of African educational experience. As noted in the Introduction, this vantage point means the paper's claims are strongest for Anglophone Southern African contexts and are offered more tentatively, as hypotheses rather than findings, for Francophone, Lusophone, and North African higher education systems, whose colonial and linguistic histories differ in ways that could substantially change how the argument's five principles would need to be operationalized. The paper draws primarily on scholarship in English, which itself reflects the linguistic hierarchies that operate within global academic publishing and that constrain the circulation of knowledge produced in African languages. Acknowledging these limitations is part of what epistemic honesty in the decolonial project demands.

## Mechanisms of epistemic injustice in African higher education assessment

### The standardization imperative and its exclusions

The dominant assessment framework in African higher education continues to be organized around an imperative of standardization that was institutionalized during the colonial period and has proved remarkably durable in the postcolonial era. Standardization—the requirement that all students be evaluated against the same criteria, using the same formats, within the same time constraints was designed to serve the values of objectivity, comparability, and meritocracy. In practice, however, standardization as currently implemented in most African universities presupposes a particular kind of learner: one who has been adequately socialized into the norms of Western academic written discourse, who has sufficient access to the linguistic and cultural capital required to succeed in formal examination contexts, and whose prior knowledge aligns with the content frameworks prescribed by Eurocentric curricula (Ndofirepi and Gwaravanda, 2019; Steele et al., 2024).

For students whose educational backgrounds are rooted in different epistemic traditions—whose knowledge has been formed through oral transmission, through apprenticeship, through community practice, through engagement with local environmental and social realities—this standardization constitutes a systematic hermeneutical injustice. Their genuine knowledge and capabilities are not measured but obscured, because the assessment instrument is not designed to recognize the forms in which that knowledge is held and expressed. The consequence is not merely that these students perform less well than their better-prepared peers; it is that the assessment system communicates something false about their knowledge, namely, that they do not have it. This is the specific form that hermeneutical injustice takes in the assessment context: a systematic misrepresentation of epistemic reality that works in favor of those whose knowledge happens to align with the dominant format and against those whose knowledge does not.

### The language of assessment and epistemic exclusion

The language of assessment is one of the most powerful mechanisms through which epistemic injustice is produced and reproduced in African higher education. The overwhelming majority of formal assessment in African universities is conducted in European colonial languages—English, French, or Portuguese- which function as the languages of academic instruction across the continent despite being the first languages of relatively small minorities of students. The use of a colonial language as the exclusive medium of assessment creates a compounded disadvantage for students whose first languages are African: they must simultaneously demonstrate subject knowledge and perform in a language that is not their mother tongue, under time pressure, in high-stakes contexts (Ndlovu-Gatsheni, 2021; Santos, 2018).

The problem is not simply one of linguistic proficiency, though that is significant enough. It is also a problem of epistemic translation: many concepts, relationships, and ways of understanding the world that are deeply embedded in African languages and knowledge traditions have no adequate equivalents in English or French. When students are required to express their knowledge only in a colonial language, they are in effect required to leave behind the conceptual frameworks through which much of their knowledge is organized. What is lost in this translation is not merely stylistic; it is substantive. The assessment system registers this loss as a failure of knowledge, when in reality it is a failure of the assessment framework to provide adequate channels through which knowledge can be communicated. This is a hermeneutical injustice in the precise sense that Fricker (2007) describes: a structural gap in the interpretive resources of the assessment system that makes certain forms of knowledge systematically invisible.

A promising, and increasingly evidence-based, response to this specific problem is translanguaging in assessment: allowing students to draw flexibly on their full linguistic repertoire, moving between English, French, or Portuguese and one or more African languages within a single response, rather than being confined to a single, externally imposed medium (Ngubane, 2026). Empirical work on multilingual university assessment in South Africa demonstrates that this is not merely a normative aspiration but an implementable practice, with documented approaches including bilingual glossaries and terminology support, code-switching permitted in oral and written responses, and assessment rubrics that credit conceptual accuracy independently of which language it is expressed in Antia, 2017. We return to the practical challenge this raises for linguistically heterogeneous classrooms, where a single class may contain speakers of a dozen or more languages, in the discussion of linguistic justice below.

### The devaluation of indigenous knowledge forms

Beyond language, conventional assessment frameworks in African higher education tend to privilege specific epistemic forms that are themselves culturally particular: the written argumentative essay, the structured examination response, the individual project or thesis, the literature review that positions research findings within an established body of published academic scholarship. Each of these formats encodes assumptions about how knowledge is produced and communicated that are not universally shared. In many African knowledge traditions, knowledge is produced collaboratively rather than individually, transmitted orally rather than in writing, and validated through community practice and consensus rather than through peer review by distant experts (Chilisa, 2020; Asante, 2003).

The integration of indigenous knowledge systems into African education has been a persistent scholarly and policy concern since independence, yet its actualisation in assessment practice remains limited and uneven. A recent South African study of teacher education curricula found that, even where Indigenous Knowledge Systems have been formally written into curriculum policy, their translation into actual assessment criteria and marking practice lags well behind their inclusion in course content, with lecturers reporting uncertainty about how to evaluate indigenous knowledge rigorously within conventional grading structures (Makokotlela and Gumbo, 2025). Oral traditions—the elaborate systems of knowledge preservation and transmission through storytelling, proverb, song, and ritual that have sustained communities across the continent- are rarely acknowledged as legitimate forms of evidencing academic knowledge. Experiential and apprenticeship learning, through which practical, technical, and relational knowledge is transmitted across generations within communities, finds no place in credit-bearing assessment frameworks. Communal validation processes, through which knowledge claims are tested through dialogue and contestation within communities of practice rather than through individual performance, have no formal status in institutional assessment (Fataar and Subreenduth, 2015; Chilisa, 2020). The result is that assessment systems in African universities implicitly communicate, repeatedly and systematically, that the knowledge forms characteristic of African cultural traditions are epistemically inferior to those of the Western academy—a communication that constitutes a form of epistemic violence in the sense that Santos (2018) calls epistemicide.

### Assessment as a colonial inheritance

It is important to recognize that the epistemic injustices described above are not accidental features of assessment systems that happen to be poorly designed; they are structural features of systems that were deliberately designed to serve colonial purposes. Formal assessment in African universities was introduced as a mechanism for selecting and credentialling a class of African administrators and professionals who could function within colonial bureaucracies, which required, above all, that they could demonstrate competence in European languages, familiarity with European knowledge, and compliance with European epistemic norms (Ndlovu-Gatsheni, 2021; Mignolo, 2012). The persistence of these assessment frameworks in the postcolonial university is not a failure of reform but a consequence of the deep embeddedness of colonial institutional logics in structures that were not fundamentally reformed at independence.

Hungwe and Ndofirepi (2022) argue that meaningful decolonization of higher education requires engaging not only with the content of curricula but with the institutional and procedural dimensions of universities, including, crucially, the assessment frameworks that determine what counts as credentialled knowledge. Without assessment reform, curriculum decolonization remains incomplete and, in some respects, cosmetic: students may encounter more African content and more African perspectives in their courses, but they will still be evaluated through instruments that measure their ability to reproduce Eurocentric forms of academic performance rather than to demonstrate the full range of their epistemically diverse capabilities.

## Indigenous knowledge systems and Afrocentric alternatives for assessment

### Oral traditions and the assessment of communicated knowledge

African oral traditions represent one of the most sophisticated systems of knowledge preservation, transmission, and validation that humanity has developed. The griotic traditions of West Africa, the isibongo praise poetry traditions of Southern Africa, the oral historiography of communities across the continent—these are not merely aesthetic or cultural practices but epistemological ones: systems through which knowledge is held, tested against experience, refined through dialogue, and transmitted across generations with remarkable fidelity and sophistication (Chilisa, 2020; Asante, 2003). Yet these traditions find no place in conventional academic assessment, which requires the demonstration of knowledge through written text rather than through spoken performance, narrative, or dialogue.

Incorporating oral modes of assessment such as oral examinations, structured academic discussions, performance-based demonstrations, and storytelling as a form of knowledge presentation would not only broaden the range of competencies that assessment can capture; it would also draw on and legitimate modes of knowledge communication that are deeply familiar to many African students. This is not merely a hypothetical proposal. A documented case study of a history course at a South African university shows that replacing a conventional written examination with historical plays, songs, and reflective performance-based assessment enabled students to connect personal and community histories to academic content and, according to the reported outcomes, deepened engagement without a loss of assessed rigor, though evaluators had to develop new marking criteria appropriate to spoken and performed work (Godsell, 2021; Godsell et al., 2024). The challenge is not that oral assessment is unrigorous but that it requires different frameworks of evaluation: frameworks that assess the quality of reasoning, the depth of understanding, and the relevance of knowledge to context in ways that are appropriate to spoken rather than written performance. Developing such frameworks is a scholarly task that requires serious engagement with indigenous epistemic traditions rather than merely the substitution of one form of assessment for another within an unchanged evaluative philosophy.

### Communal and participatory validation of knowledge

Many African philosophical traditions emphasize the communal and relational dimensions of knowledge. The concept of Ubuntu, often rendered as “I am because we are,” encodes a fundamentally relational ontology in which personhood, knowledge, and capability are understood as constituted through relationships with others rather than as properties of isolated individuals (Hungwe and Ndofirepi, 2022; Gasa and Kpum, 2026). From an Ubuntu-informed perspective, the standard model of individual assessment, in which each student must demonstrate knowledge independently, without the assistance of peers, community, or collaborative dialogue, is not merely technically limiting but philosophically misaligned with the epistemological framework within which many African students understand the nature of knowledge itself.

Participatory and communal forms of assessment, group projects evaluated through processes of collective presentation and critique, community-engaged assessments in which students' knowledge is validated through application to real community problems, portfolio assessments that document knowledge developed through sustained engagement with community contexts are more congruent with these epistemological commitments. Group portfolio assessment implemented across several South African universities offers one such documented example: students collaboratively compiled and reflected on shared bodies of work, with the reported effect of sustaining peer dialogue and mentoring and shifting assessment from an individually competitive exercise toward a co-constructed one, though the researchers also noted the practical burden of designing marking criteria that could still distinguish individual contribution within a genuinely collaborative product (Fouche et al., 2021). Chilisa (2020) argues that indigenous research and evaluation methodologies that draw on community participation not only produce richer knowledge but are also more ethically appropriate when the communities being studied or served are themselves the primary audience for the knowledge produced. The same argument applies to assessment: an assessment system that involves community members in defining what counts as competent performance, that values knowledge demonstrated through community engagement, and that treats the community as a legitimate site of epistemic validation rather than merely as a context for individual learning is more epistemically just than one that confines assessment to individual performance within institutional walls.

### Experiential knowledge and ubuntu-based evaluation

The concept of experiential learning, knowledge derived from direct engagement with the world through practice, observation, and reflection, has a long history within progressive educational theory (Kolb, 1984). Within African indigenous educational traditions, experiential knowledge has always occupied a central place: apprenticeship systems through which young people learn agricultural, medicinal, crafting, and social knowledge through sustained community participation; initiation processes through which cultural knowledge and communal values are transmitted through embodied experience; and problem-based communal learning in which knowledge is developed through collective engagement with practical challenges. These forms of knowledge production and validation are not methodologically inferior to those of formal academic education; they are differently organized and oriented toward different ends.

Assessment frameworks that can recognize and credit experiential learning would represent a significant step toward epistemic justice in African higher education. The African Rural University (ARU) in Uganda offers perhaps the most fully realized institutional example currently documented in the literature. ARU structurally embeds Indigenous Knowledge Systems throughout its curriculum and assessment model, employing Traditional Wisdom Specialists alongside conventional academic instructors, and organizes its programe around roughly 40% practical, community-embedded learning, including herbal medicine, indigenous agriculture, and participatory development practice, assessed through demonstrated competence and community-facing outputs rather than written examination alone. Graduates are credentialled as Rural Transformation Specialists and go on to serve as change agents within the communities where their competence was originally demonstrated and validated (Maali, 2025). The case is not offered as a template that transfers unchanged to other institutional settings; ARU is a small, mission-specific institution, and its model would need substantial adaptation for large, multi-faculty research universities operating under conventional national accreditation frameworks. It is offered instead as evidence that experiential, community-validated assessment at scale is institutionally possible rather than only theoretically desirable. This might include the formal recognition of prior learning acquired in community contexts, the integration of community-based fieldwork as assessed components of academic programmes, and the development of assessment criteria that are sensitive to the practical, relational, and contextual dimensions of knowledge rather than only to its propositional, text-based expression. Boadu and Ile (2023) argue that what they call “indigenously responsive evaluation”—evaluation that is grounded in the knowledge traditions and validation processes of local communities—is not only more equitable but more likely to generate knowledge that is actually useful for community development, precisely because it recognizes the community as a legitimate site of both knowledge production and knowledge evaluation.

## Digital and AI-based assessment: risks and responsibilities

### The promise and the peril of algorithmic assessment

The growing deployment of digital and AI-based assessment tools in higher education has generated considerable enthusiasm, particularly in contexts where teacher shortages and large student populations create pressure to find scalable alternatives to human marking. Automated essay scoring, intelligent tutoring systems, learning analytics, and AI-powered adaptive assessment platforms (including automated essay-scoring engines embedded in learning management systems, AI-based plagiarism and “AI-writing” detection tools now routinely applied to student submissions, and adaptive testing platforms used in some large-enrolment gateway courses) are being actively promoted across African higher education systems as solutions to problems of assessment quality and consistency. There is genuine reason to take these tools seriously as educational innovations; when well-designed and appropriately deployed, they can provide timely feedback, identify learning gaps, and reduce the administrative burden on educators.

However, the deployment of AI-based assessment in African contexts carries specific epistemic justice risks that have received insufficient attention in the policy discourse surrounding educational technology. AI assessment systems are trained on large datasets that reflect the historical output of particular student populations—populations that, in most cases, are drawn disproportionately from high-resource, English-language, Eurocentric academic contexts. The patterns that these systems learn to recognize and reward as indicators of academic quality are therefore patterns that are characteristic of students who have been successfully socialized into Western academic norms (Pasipamire and Muroyiwa, 2024). When these systems are deployed to assess students whose writing, argumentation, and knowledge expression differ from those of the training population, as will frequently be the case in African universities, they are liable to produce systematically biased evaluations that penalize culturally different but epistemically legitimate forms of academic work. It should be acknowledged directly that rigorous, published empirical studies quantifying this bias specifically among African student populations remain scarce; the argument here is a reasonable extrapolation from documented algorithmic bias in comparable contexts elsewhere, combined with the general findings on training-data skew reported by Pasipamire and Muroyiwa (2024), rather than a claim resting on a settled body of African-specific evidence. This is itself a research gap the paper's fifth principle, below, is intended to help close.

### Digital colonialism and epistemic technology

The risks of AI-based assessment in Africa cannot be understood in purely technical terms; they require a political analysis of the global structures of technology production and deployment. Pasipamire and Muroyiwa (2024) argue that algorithmic bias in the African context is not merely a technical problem amenable to data engineering solutions, but an expression of the broader structures of digital colonialism through which technologies developed in and for the Global North are exported to the Global South in ways that embed Northern cultural values and knowledge hierarchies into the digital infrastructure of education. AI assessment tools that encode Eurocentric evaluation standards are not epistemically neutral instruments; they are epistemic technologies—tools that reproduce and reinforce particular conceptions of what counts as knowledge and competent academic performance. Digital access alone does not guarantee epistemic inclusion: even formally enrolled, digitally connected distance learners can remain relationally and culturally excluded from the informal networks that support academic success, a dynamic documented among African Open and Distance e-Learning students (Kpum and Gasa, 2026a). When African students are assessed by these tools, they encounter, once again, the hermeneutical injustice that has characterized assessment in African education since the colonial period: an evaluative framework that was not designed to recognize the forms of knowledge they hold.

Continental and international policy has begun, tentatively, to engage this problem. The African Union's Continental Artificial Intelligence Strategy, adopted in 2024, commits member states to “people-centric, development-oriented and inclusive” AI governance, with an implementation plan running to 2030 (African Union, 2024). UNESCO's Recommendation on the Ethics of Artificial Intelligence, adopted in 2021 and since taken up by 29 African states in national strategy development, similarly frames algorithmic fairness and cultural inclusion as governance obligations rather than optional design considerations (UNESCO, 2021), and UNESCO's subsequent AI Competency Framework for Students (Miao and Shiohira, 2024) sets out human-centered, ethics-first expectations for how AI tools should be introduced into teaching and assessment. None of these instruments, however, contains binding, assessment-specific standards for how AI scoring systems deployed in African universities should be validated against African student populations before use; they establish a normative direction rather than an enforceable floor. This gap between high-level policy commitment and assessment-specific technical accountability is precisely what the paper's fifth principle, below, is intended to address.

A genuinely decolonial approach to AI assessment in Africa would require, at minimum, the following: the development of training datasets that include the full linguistic and cultural diversity of African students' academic output; the involvement of African educators, communities, and students in the design of assessment criteria and the validation of AI scoring systems against locally defined standards of academic quality; and the establishment of accountability mechanisms that enable communities to challenge AI assessment decisions that they experience as epistemically unjust. These requirements are demanding and, in the current state of the field, largely unmet. The risk, as Boadu and Ile (2023) observes, is that Euro-American assessment approaches, including AI-based tools, will continue to be imported into African educational contexts without adequate contextual adaptation, reproducing the epistemic exclusions of colonial assessment at a larger scale and with greater technological authority.

## Toward epistemically just assessment in African higher education

### Principles for decolonized assessment practice

**Figure 1:**
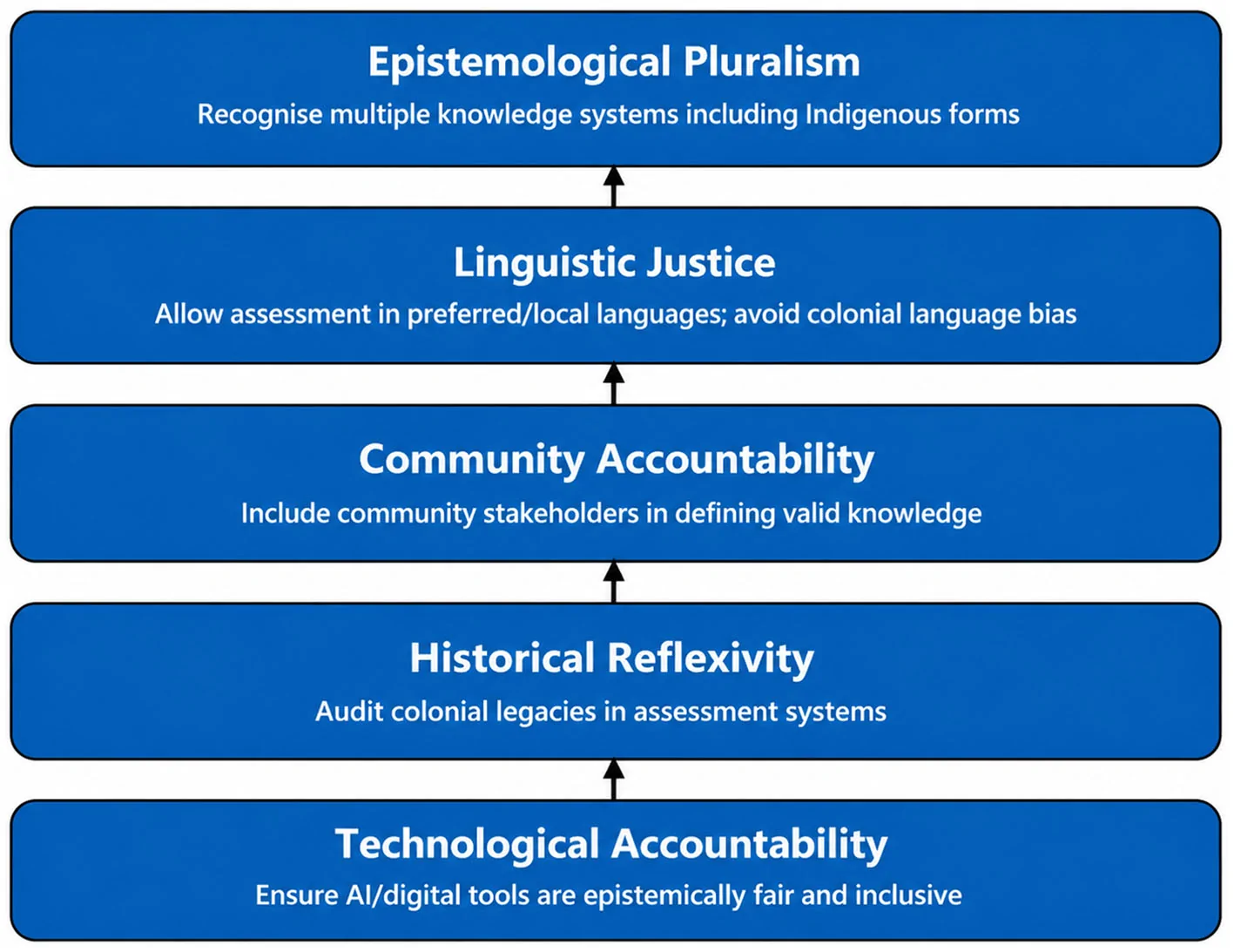
A networked, non-hierarchical model of the five principles of epistemically just assessment. Each principle is connected to every other by bidirectional arrows rather than a single vertical sequence, reflecting the argument, made explicit above, that no principle is foundational to or superior to the others; progress on any one principle (for example, technological accountability) is intended to reinforce, and be reinforced by, progress on the others (for example, epistemological pluralism), rather than serving merely as a base layer beneath them.

Drawing together the theoretical analysis presented above, this paper proposes five interconnected and, deliberately, non-hierarchical principles for epistemically just assessment in African higher education. The principles are presented in sequence below for expository clarity, but that sequence is not a ranking: no principle is foundational to or more important than the others, and Figure 1 represents them accordingly as a networked cycle rather than a hierarchy. These principles do not constitute a complete reform programe but a set of normative orientations that should guide assessment design, institutional policy, and professional development for educators across the continent.

The first principle is epistemological pluralism: assessment systems should be designed to recognize and credit knowledge that is expressed through multiple epistemological frameworks, including those rooted in African indigenous traditions. This requires moving beyond the assumption that there is a single valid mode of academic knowledge—written, individual, language-in-English- and developing evaluation frameworks that can engage with oral, communal, experiential, and vernacular forms of knowing as epistemically legitimate.

The second principle is linguistic justice: assessment systems should provide meaningful opportunities for students to demonstrate knowledge in their first or preferred languages, rather than requiring all knowledge to be expressed through a colonial language. This does not require the abandonment of multilingual academic communication, but it does require that linguistic ability in a colonial language not be allowed to function as a proxy for knowledge of the assessed subject matter. Operationalizing this principle in the typical African university lecture hall, where a single class may contain speakers of a dozen or more first languages, cannot mean offering a separate assessment instrument for every language represented; that is rarely administratively feasible. A more workable path, supported by emerging South African evidence, is translanguaging pedagogy and assessment: permitting students to draw on the full range of their linguistic repertoire within a single response, supporting this with bilingual glossaries and terminology support, and training markers to assess conceptual and disciplinary accuracy independently of which language or combination of languages it is expressed in Ngubane (2026) and Antia (2017). This shifts linguistic justice from an all-or-nothing choice between a colonial language and an indigenous one toward a flexible, resourced practice that individual departments can pilot at manageable scale.

The third principle is community accountability: assessment frameworks should involve the communities served by higher education institutions in defining what counts as competent, relevant, and valuable knowledge. Community members, traditional knowledge holders, and local experts should have legitimate voice in assessment design, alongside academic specialists, and assessment outcomes should be evaluated not only by academic criteria but by their relevance and usefulness to community development.

The fourth principle is historical reflexivity: assessment systems should be evaluated not only for their technical properties but for their historical trajectory—for how they carry forward, or challenge and transform, the epistemic hierarchies that were established during the colonial period. This requires a sustained commitment by institutions to audit their assessment practices for colonial inheritances and to develop deliberate reform strategies that are oriented toward epistemic liberation rather than merely toward technical improvement.

The fifth principle is technological accountability: the deployment of digital and AI-based assessment tools in African higher education should be subject to rigorous epistemic justice evaluation before implementation. This means requiring developers to demonstrate that their systems have been trained on and validated against data that adequately represents the diversity of African students, and that their evaluative criteria are not calibrated exclusively against Eurocentric standards of academic quality.

Figure 1 below: A Networked Model of Epistemically Just Assessment in African Higher Education, with the Five Principles Arranged as Mutually Reinforcing Nodes Rather Than a Hierarchy.

This five-principle framework is developed alongside a parallel, more general account of assessment justice—organized around distributive, recognitional, participatory, representational, and restorative dimensions—that Kpum and Gasa (2026b) have elaborated elsewhere; the two frameworks are complementary, with the present paper's principles specifying what recognitional and participatory justice require in the specific case of Indigenous Knowledge Systems and Afrocentric epistemology.

### Navigating the tension with international comparability and graduate mobility

A serious objection to the argument developed here is that African universities do not operate in an institutional vacuum: they are embedded in global quality assurance architectures, professional body accreditation requirements, and graduate labor markets that often mandate standardized, English-medium assessment as a condition of degree recognition, professional licensure, or admission to postgraduate study abroad. A student assessed through an epistemically pluralistic, community-validated, oral, or translanguaged process may find that credential difficult to “translate” for an international accreditation body, an overseas graduate admissions committee, or a regional professional council operating under frameworks such as those coordinated through the African Quality Assurance Network (AQAN). This tension is real, and a paper proposing assessment reform has an obligation to say more about it than that institutions should “navigate” it.

Three partial strategies are worth naming, none of which fully resolves the tension but each of which offers African institutions a workable middle path. The first is dual-track or layered assessment, in which epistemically pluralistic methods (oral defense, portfolio, and community-validated project work) run alongside, rather than replacing, a standardized core sufficient to satisfy external accreditation, with the pluralistic component certified as additional evidence of competence rather than a substitute credential; this preserves comparability for the credential that travels while still expanding what counts within the institution's own teaching and learning practice. The second is engagement with regional qualification-recognition frameworks themselves, since bodies such as AQAN and the African Union's own qualifications-recognition initiatives are, in principle, sites where African institutions collectively can press for portfolio- and competence-based equivalencies to be recognized as equal in standing to written examination, rather than accepting standardization as an externally fixed constraint; this is slower and more political than a single institution's assessment redesign, but it is the level at which the tension is ultimately negotiable. The third is transparent equivalency documentation: where a student is assessed through an oral, communal, or translanguaged method, the institution issues a clear, internationally legible statement of the competencies demonstrated and the method used, so that a receiving institution or employer can evaluate the credential on its stated merits rather than discounting it by default for not conforming to a written, individual, English-medium norm. None of these strategies eliminates the risk that epistemically just assessment could, in the short term, cost some graduates international legibility; that risk is a genuine cost of the reform this paper argues for, and honesty about it is preferable to implying the tension away.

### Practical implications for assessment design

These principles have concrete implications for assessment practice. They suggest, among other things, the systematic expansion of oral and performance-based assessment components in African university programes; the formalization of community-based assessment where students demonstrate knowledge through engagement with community projects and are evaluated partly by community members; the recognition of prior learning in indigenous and community contexts for credit; the development of multilingual assessment options where feasible, building on translanguaging approaches already piloted in South African higher education (Ngubane, 2026); and the reform of marking rubrics to include criteria that recognize culturally diverse modes of argumentation, knowledge presentation, and epistemic reasoning.

At the institutional level, these implications point to the need for sustained professional development of academic staff in culturally responsive assessment theory and practice; the review of accreditation and quality assurance frameworks to remove barriers to assessment innovation; and the investment in research on the relationship between assessment practices and the epistemic experiences of students from diverse African communities. None of these changes will be achieved easily or quickly; they require genuine institutional commitment and the willingness of universities to challenge the accreditation and quality assurance frameworks that have historically constrained assessment reform in the name of international comparability.

## Conclusion

This paper has argued that epistemic injustice in educational assessment is not a peripheral problem of technique but a central problem of the politics of knowledge. The assessment systems that govern how student learning is evaluated in the Anglophone, predominantly Southern African higher education contexts examined here were designed within and for Eurocentric epistemological traditions, and they continue to reproduce the hierarchical assumptions of those traditions in ways that marginalize indigenous knowledge systems, Afrocentric ways of knowing, and the culturally diverse epistemic repertoires that African students bring with them to university. Drawing on Fricker's theory of epistemic injustice, Santos's ecology of knowledges, Ndlovu-Gatsheni's concept of the cognitive empire, and Asante's Afrocentric paradigm, and grounding the discussion in documented institutional cases from South Africa and Uganda, the paper has traced the specific mechanisms—standardization, colonial language requirements, the devaluation of indigenous knowledge forms, and the reproduction of colonial institutional logic- through which this injustice operates.

The paper has further argued that emerging digital and AI-based assessment tools, far from offering a neutral solution to these problems, risk amplifying and entrenching them unless they are developed and deployed in ways that are explicitly responsive to the epistemic diversity of African students. The deployment of AI assessment tools that embed Eurocentric evaluative standards in algorithmic form represents a new iteration of a very old problem: the technological enforcement of epistemic hierarchy at scale.

Against this backdrop, the paper has proposed five mutually reinforcing, non-hierarchical principles—epistemological pluralism, linguistic justice, community accountability, historical reflexivity, and technological accountability- as the normative foundation for epistemically just assessment in the African higher education contexts on which this paper has focused. These principles are not aspirational rhetoric but practical requirements for assessment systems that genuinely serve all learners, even as their translation into practice must reckon honestly with the real tension between locally responsive assessment and the international accreditation systems within which African universities operate. Meeting them will require not only technical innovation in assessment design but a willingness on the part of African universities to confront honestly the colonial inheritance that continues to shape how knowledge is measured, validated, and credentialled within their walls. The project of epistemic justice in assessment is, ultimately, inseparable from the broader project of decolonizing African higher education, and both projects are urgently necessary, and the paper's Southern African vantage point is offered as one contribution to a wider, necessarily comparative conversation rather than as its final word.
